# SVM-Based Optical Detection of Retinal Ganglion Cell Apoptosis

**DOI:** 10.3390/photonics12020128

**Published:** 2025-01-31

**Authors:** Mukhit Kulmaganbetov, Ryan Bevan, Andrew Want, Nantheera Anantrasirichai, Alin Achim, Julie Albon, James Morgan

**Affiliations:** 1 School of Optometry and Vision Sciences, Cardiff University, Cardiff CF24 4HQ, UK; 2 Glaucoma Department, Kazakh Eye Research Institute, Almaty A05H2A8, Kazakhstan; 3 Centre for Eye and Vision Research (CEVR), 17W Hong Kong Science Park, Hong Kong; 4 UK Dementia Research Institute, Cardiff University, Cardiff CF24 4HQ, UK; 5 Visual Information Laboratory, University of Bristol, Bristol BS8 1TH, UK

**Keywords:** modulation transfer function, optical coherence tomography, retinal ganglion cell, support vector machine, principal component analysis

## Abstract

**Background:**

Retinal ganglion cell (RGC) loss is crucial in eye diseases like glaucoma. Axon damage and dendritic degeneration precede cell death, detectable within optical coherence tomography (OCT) resolution, indicating their correlation with neuronal degeneration. The purpose of this study is to evaluate the optical changes of early retinal degeneration.

**Methods:**

The detection of optical changes in the axotomised retinal explants was completed in six C57BL/6J mice. OCT images were acquired up to 120 min from enucleation. A grey-level co-occurrence-based texture analysis was performed on the inner plexiform layer (IPL) to monitor changes in the optical speckles using a principal component analysis (PCA) and a support vector machine (SVM). In parallel tests, retinal transparency was confirmed by a comparison of the modulation transfer functions (MTFs) at 0 and 120 min.

**Results:**

Quantitative confirmation by analysis of the MTFs confirmed the non-degradation of optical transparency during the imaging period: MTF (fx) = 0.267 *±* 0.02. Textural features in the IPL could discriminate between the optical signals of RGC degeneration. The mean accuracy of the SVM classification was 86.3%; discrimination was not enhanced by the combination of the SVM and PCA (81.9%).

**Conclusions:**

Optical changes in the IPL can be detected using OCT following RGC axotomy. High-resolution OCT can provide an index of retinal neuronal integrity and its degeneration.

## 1 Introduction

Retinal ganglion cell (RGC) loss is a signature pathological event in many eye diseases and remains a topic of intense study [1–4]. Axon damage is a common driver of these changes in conditions such as glaucoma and traumatic optic neuropathy in which a compromised axonal transport initiates the disruption of organelles such as mitochondria [5,6] as a prelude to the initiation of programmed cell death. This sequence of neuronal degeneration provides a useful temporal window for detecting RGC dysfunction before the onset of cell death.

Dendritic degeneration has emerged as a robust marker of RGC degeneration [7]. Dendrite pruning can be driven extracellularly by the activation of the complement cascade pathway [8] and intracellularly through caspase activation [9–11]. Common to both is the fragmentation of the mitochondrial network, a characteristic feature of pre-apoptotic damage that can be seen histologically as beading in RGC dendrites [12,13]. Critically, these structural changes (within the range of 1–5 µm) lie within the detection resolution of high-resolution optical coherence tomography (OCT) [14,15]. While this does not imply the optical resolution of subcellular structures, it suggests that these changes generate optical signals that correlate with neuronal degeneration [16]. The feasibility of this approach is helped by the concentration of RGC dendrites within the inner plexiform layer (IPL), which represents a practical region of interest (ROI) for the assessment of RGC degeneration (or its inverse, neuronal health). Furthermore, the higher refractive index of membranous organelles such as mitochondria (1.48) [17,18] relative to the whole cell (1.38) [19] suggests that they contribute significantly to light scattering within the IPL [20].

We have previously reported that changes in optical texture correlate with the time course of degeneration in RGC5 cells [21]. A potential confounder of this analysis is that culture-related optical transparency reductions could generate spurious degeneration signals. We aim to develop optic tools for characterising retinal neuronal degeneration, specifically focusing on the tissues that remain transparent and healthy under clinical observation. To achieve this, we evaluated these changes in a retinal explant model. Additionally, we assessed retinal transparency by measuring the modulation transfer function during an imaging interval conducive to detecting early retinal degeneration.

## 2 Materials and Methods

### 2.1 Tissue Preparation

All experiments were conducted in accordance with Home Office regulations and the ARVO Statement for the Use of Animals in Ophthalmic and Vision Research. C57BL/6J mice (minimum age of 15 months) were sacrificed by cervical dislocation (Schedule 1 Appropriate Methods of Humane Killing, United Kingdom Animal Scientific Procedures Act 1986). The number of animals used in this study is in line with the UK NC3Rs policy regarding the use of animals in research.

**Figure 1:**
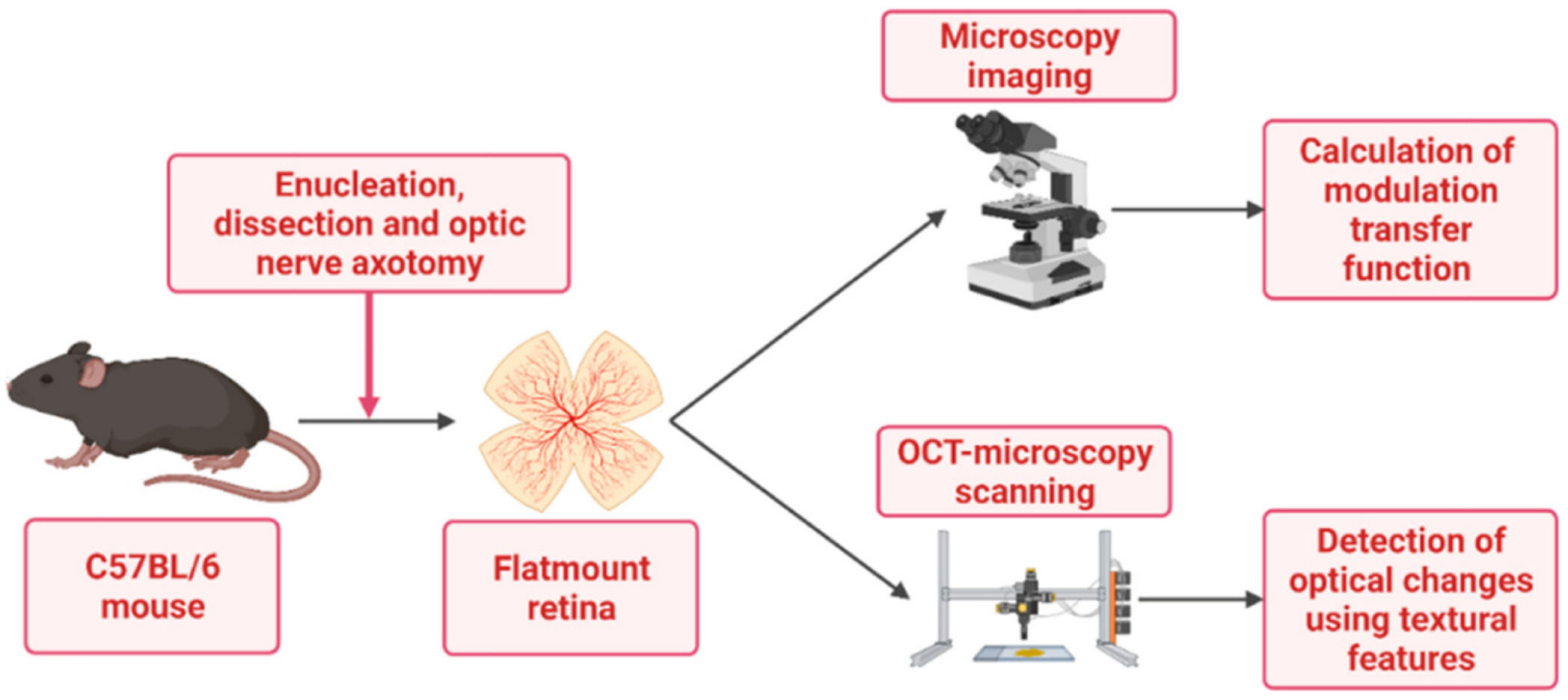
Mouse retinal explant preparation: after dissection and sectioning of the optic nerve, retinal tissue is flat-mounted in the coverslip and imaged using the Olympus-IX71 microscope (for the evaluation of transparency/opacity of retinal explants after axotomy) and OCT microscopy (for the monitoring of early RGC dendritic degeneration after axotomy). This was created with BioRender.comc.

Following enucleation, the eyes were further dissected in Hank’s balanced salt solution (HBSS, Life Technologies). The globe was penetrated at the limbus to remove the intraocular fluid, lens, and vitreous humour and to separate the retina. The retina was immediately transferred to a Petri dish and flat-mounted (ganglion cell layer up). The flat-mounted retinas were then imaged using a custom-built OCT (Figure 1), the technical details of which are described elsewhere [22]. During the post-dissection and imaging periods, the tissues were kept in a B27/Neurobasal (NB) medium (Thermo Fisher, Loughborough, UK) at room temperature. All explants were subject to the same laboratory conditions and were divided into two groups: one group for the assessment of optical transparency and the other for an OCT analysis; as it was performed in a 2 h time window, it was not technically possible to quantify both the retinal transparency and optical texture of the same sample.

### 2.2 Retinal Transparency Post Axotomy: Analysis of the Modulation Transfer Function

Explants were imaged using an Olympus-IX71 microscope to measure the modulation transfer function (MTF), computed as the normalised modulus of the fast Fourier transform (FFT) of the point spread function (PSF) [23]. The summation of the line of overlapping PSFs forms the line spread function (LSF) (Equation (1)), and we would expect the PSF width to increase if the optical transparency of the retina were compromised.

(1) $$MTF(v)=|FFT\{LSF(x)\}|=\frac{1}{\sqrt{2\pi }}\int_{-\infty }^{\infty }LSF(x)e^{-i2\pi vx}dx$$

The LSF is the first derivative of the edge spread function (ESF), the image profile of an edge [24]; the ESF is built from a set of parallel LSFs, which end at the edge position. The modulation depth of a target placed under the retina and the transfer ratios were calculated (Equation (2)) and (Equation (3)) using the pixel intensity ranges from digital images (1360 × 1024 pixels). The MTF is defined as the ratio of the image’s contrast with the tissue to the contrast of the micrograph of the calibration slide.

(2) $$MTF\left(f_{x}\right)=\frac{M_{I}\left(f_{x}\right)}{M_{O}\left(f_{x}\right)}$$

where *M*_*I*_(*f*_*x*_) and *M*_*O*_(*f*_*x*_) are the modulation depths of the images of the grid lines with and without the retinal explant, respectively. Equation (2) can be expanded as Equation (3).

(3) $$\begin{array}{c} M_{I}\left(f_{x}\right)=\frac{\left(I_{max-I}-I_{min-I}\right)}{\left(I_{max-I}+I_{min-I}\right)}\\ M_{O}\left(f_{x}\right)=\frac{\left(I_{max-O}-I_{min-O}\right)}{\left(I_{max-O}+I_{min-O}\right)} \end{array}$$

where *I*_*max−I*_ and *I*_*min−I*_ are the maximum and minimum intensity of the image with the retinal explant (*I*—image); *I*_*max−O*_ and *I*_*min−O*_ are the maximum and minimum intensity of the image without the retinal explant (*O*—object).

In order to measure the maximum and minimum intensity of the image with a retinal explant, the explanted retinal samples were placed on a Petri dish with the stage micrometre calibration slide (Muhwa Scientific, Shanghai, China) comprising a 1 mm scale of high contrast black and white lines, marking 0.01 mm divisions. At baseline, a control greyscale image was taken of the calibration slide (with an overlying Petri dish and without an overlying retinal explant) to measure the *I*_*max−O*_ and *I*_*min−O*_.

**Figure 2:**
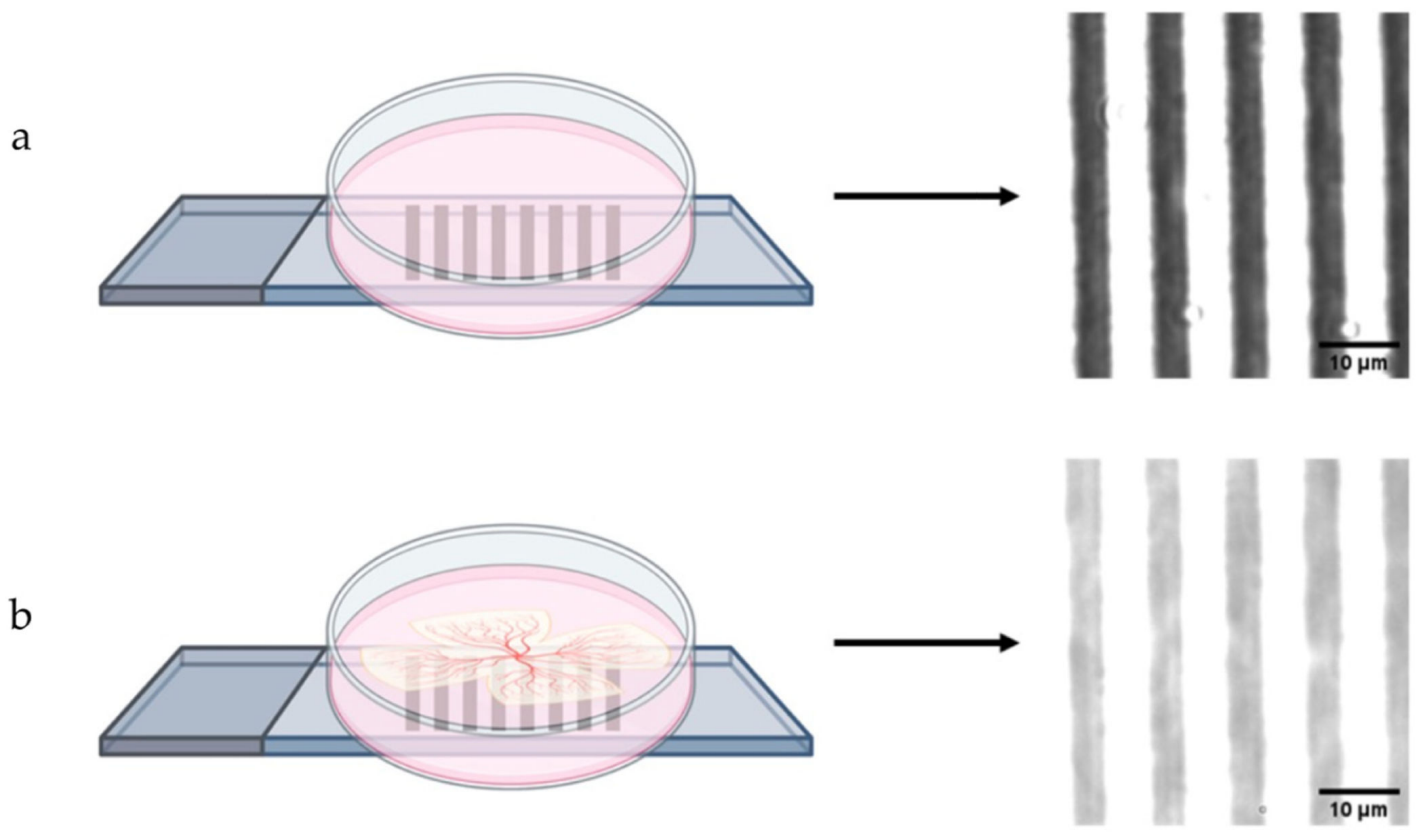
Schematic diagram of the microscopy imaging (left) and acquired micrographs (right). The control image was acquired without the explant **(a)**; the retinal tissue was placed on a Petri dish with an underlying calibration slide for the measurement of MTF in dynamics **(b)**.

Microscope images of the calibration graticule with and without the intervening retinal explant are shown in Figure 2. The *I*_*max*_ of both the images with and without the retinal tissue is 255, whereas their *I*_*min*_ differs: it is 57 for the image without an explant and 138 for the image with an explant.

### 2.3 Retinal Atrophy Post Axotomy: Texture Analysis of RGC Dendritic Tree

The dispersion compensation of the OCT images was performed during the image acquisition process, and all images were inspected as part of quality assurance before image processing. The analysis was conducted on a separate retinal series from those used for MTF analysis. The spectral data generated by OCT microscopy were processed into TIFF files with custom-built file conversion software (VSBL, Cardiff University, Cardiff, UK). An FFT filter was used to remove line-scan artefacts and background noise using FIJI Image J (NIH). The volumes of interest (VOI) and the textural features were then selected as previously reported [25].

OCTs were taken of 3 C57BL/6J mice explants (2 images from each retina) and were performed following insertion into the culture plate at the time points 0, 30, 60, and 120 min to provide 24 images. A total of 10 volumes of interest (VOI) were then manually selected within the IPL to provide 20 VOI for each retina, and 240 VOI in total for all the time points. Each VOI comprised 30 × 30 × 30 pixels (*x, y* and *z*): *x* and *z* coordinates were selected by a random number generator, and the *y* coordinate was chosen from the IPL/ganglion cell layer border along with the IPL thickness.

Five grey-level co-occurrence matrix (GLCM) features in four directions (0°, 90°, 180°, and 270°) were extracted from each VOI using the FIJI plug-in “GLCM Texture” [26,27]. GLCM features (n = 20) (energy, contrast, entropy, correlation, and homogeneity) were extracted for four spatial directions. The 20-dimensional feature space was then analysed using machine learning (ML) tools—a principal component analysis (PCA) and support vector machine (SVM) [28].

The first and second principal components in the 2-dimensional data set, including 95% of the variance, were chosen for the PCA. The motivation for using a PCA was the dimension reduction and cost-effectiveness of the algorithm [29,30]. Among the SVM algorithms, a linear kernel was used for its higher accuracy in our previous study, where a retinal subcellular, constituent-mimicking phantom classification was made [25]. ML-based optical sign detection analysis was performed in Simulink using MATLAB R2019b (MathWorks).

## 3 Results

In total, six retinal explants were used in this study, all maintained under identical laboratory conditions. Three explants were used to quantify the changes in optical transparency, and three for the OCT measurements of optic texture. Technically, acquiring the explant OCT image and MTF data in the same tissue was impossible. After each experiment, all explants (six) were optically clear under microscopic inspection.

### 3.1 Transparency of the Retinal Explants

**Figure 3:**
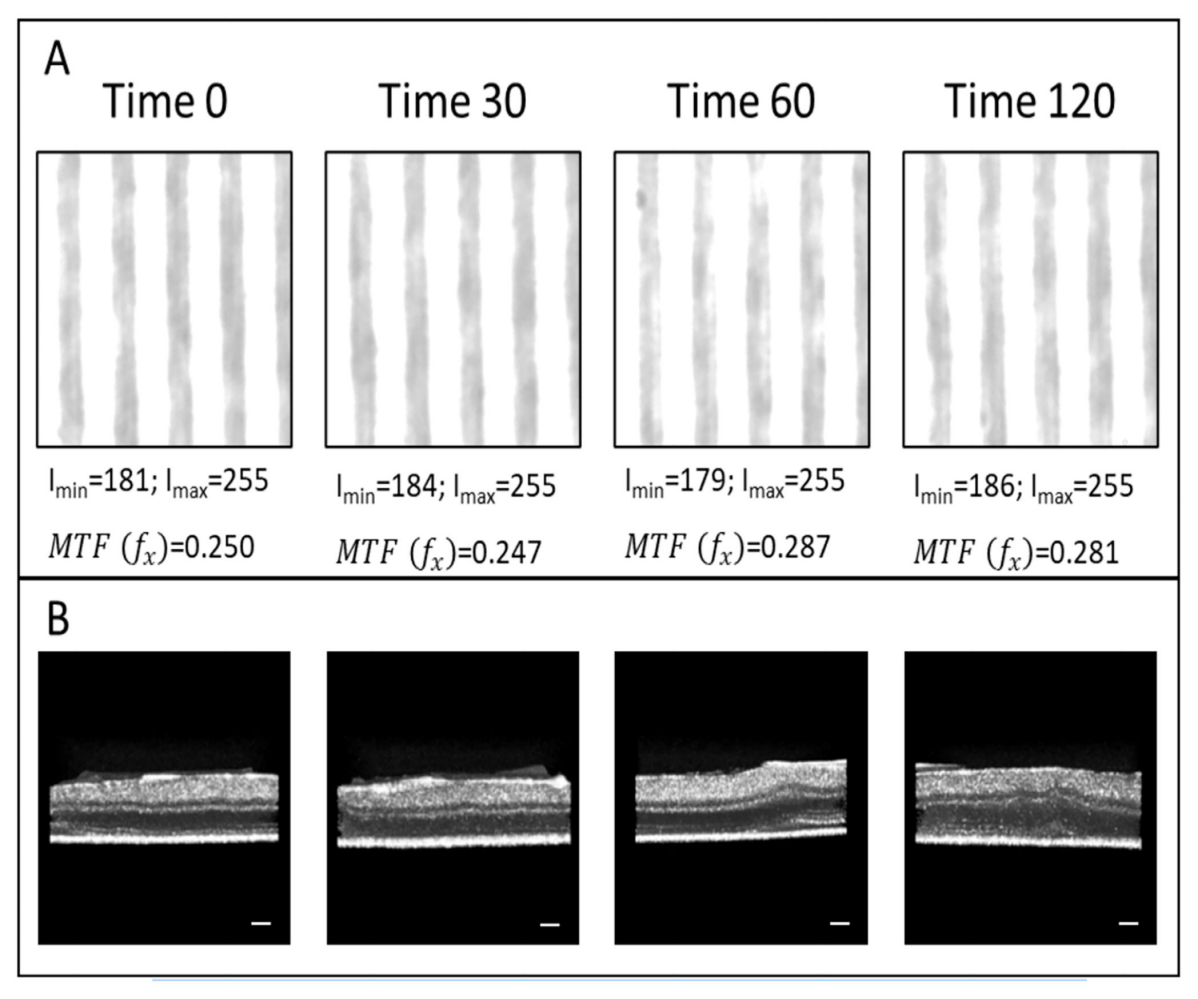
Microscopy **(A)** and OCT **(B)** images of the calibration slide with the tissue explants at different time points. The images were taken within the volumes of interest (VOI) of 30 × 30 × 30 pixels (x, y, and z). Scale bar for OCT images **(B)** = 50 µm.

Figure 3A shows the micrographs of the calibration slide with the tissue explants at different time points. The images were timed following the capture of the first image of the retina in the culture (time 0) and at 30, 60, and 120 min thereafter. The preparation time for each retinal explant was 2–3 min (*±*1.5 min), so that time 0 represented 3–4 min post-mortem. The corresponding OCT images of the murine retinal explants were acquired (Figure 3B) at the same time intervals: at 0, 30, 60, and 120 min. The samples were moved from the OCT microscope platform to replace the Petri dish lid between OCT image acquisitions, so as to prevent an exact alignment of the regions of interest due to movement in the NB medium. Since the explants were covered with the NB medium, an air–fluid interface was not discernible in the OCT images.

The mean, maximum, and minimum intensity values of the time series micrographs were measured. Using Equations (1) and (2), the modulation features were calculated and the *MTF*(*f x*) ratios are provided in Figure 3A.

**Figure 4:**
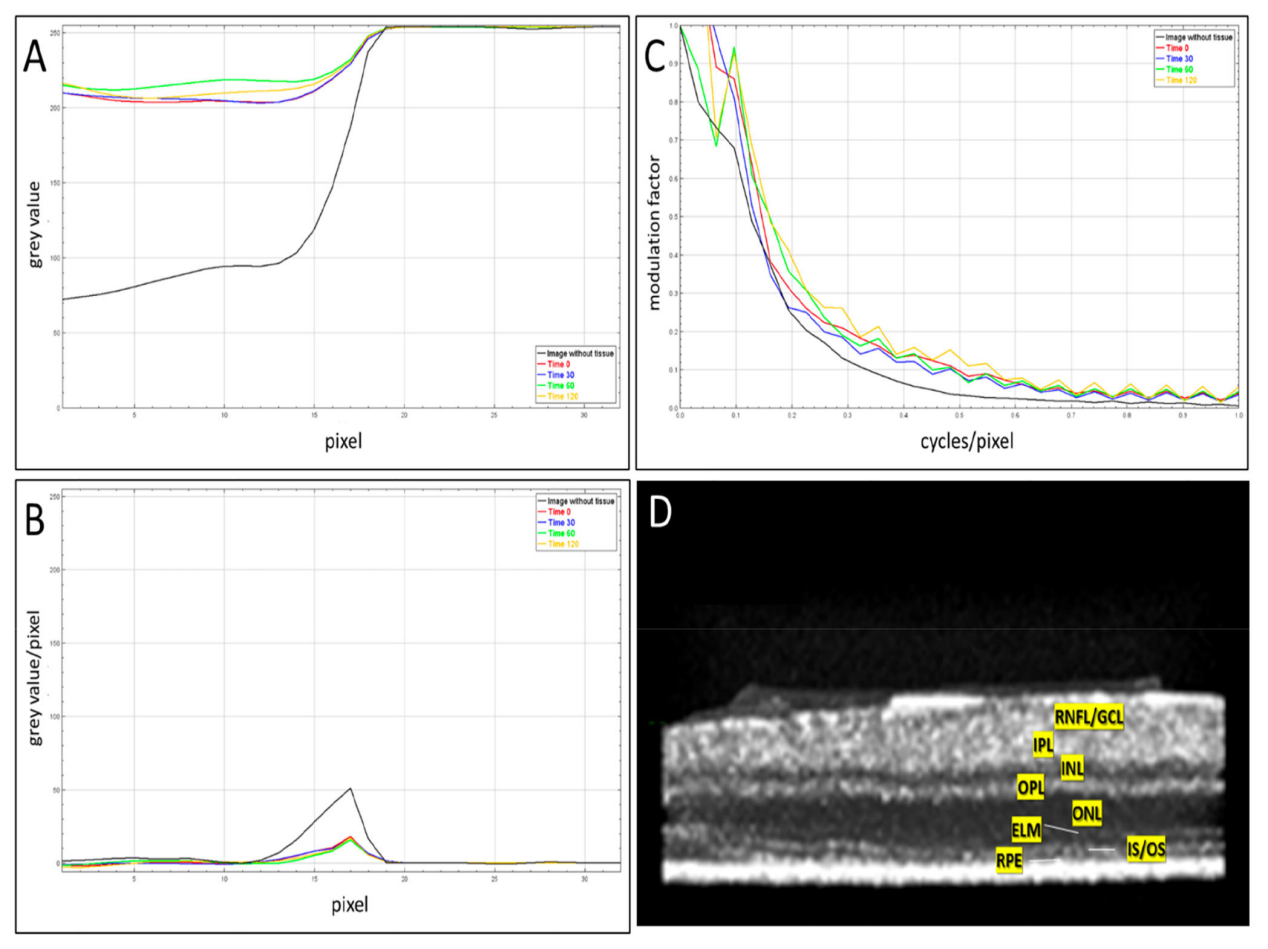
**(A)** Edge spread function, **(B)** line spread function, and **(C)** modulation transfer function for explant microscopy images with and without the retinal explants. All functions were unchanged and there were no signs of secondary opacification of tissues during the 2 h of post-dissection. **(D)** 3D rendered OCT image of a mouse retinal explant at time 0 (see Figure 3B), RGC side up. Abbreviations: RNFL—retinal nerve fibre layer; GCL—ganglion cell layer; IPL—inner plexiform layer; INL—inner nuclear layer; OPL—outer plexiform layer; ONL—outer nuclear layer; PR—photoreceptor layer; ELM—external limiting membrane; IS/OS—the junction between the photoreceptor outer and inner segments; and RPE—retinal pigment epithelium.

The ESF, LSF, and MTF relationships and the results of the computations are shown in Figure 4A–C. The x-axis of the MTF plot is the normalized modulation factor against the frequency input (cycles/pixel). The spread and modulation transfer function values remained stable, and an opacification of the retinal explants was not observed within the 2 h of imaging, confirming the preservation of optical transparency.

### 3.2 IPL Texture Analysis Post Axotomy

**Figure 5:**
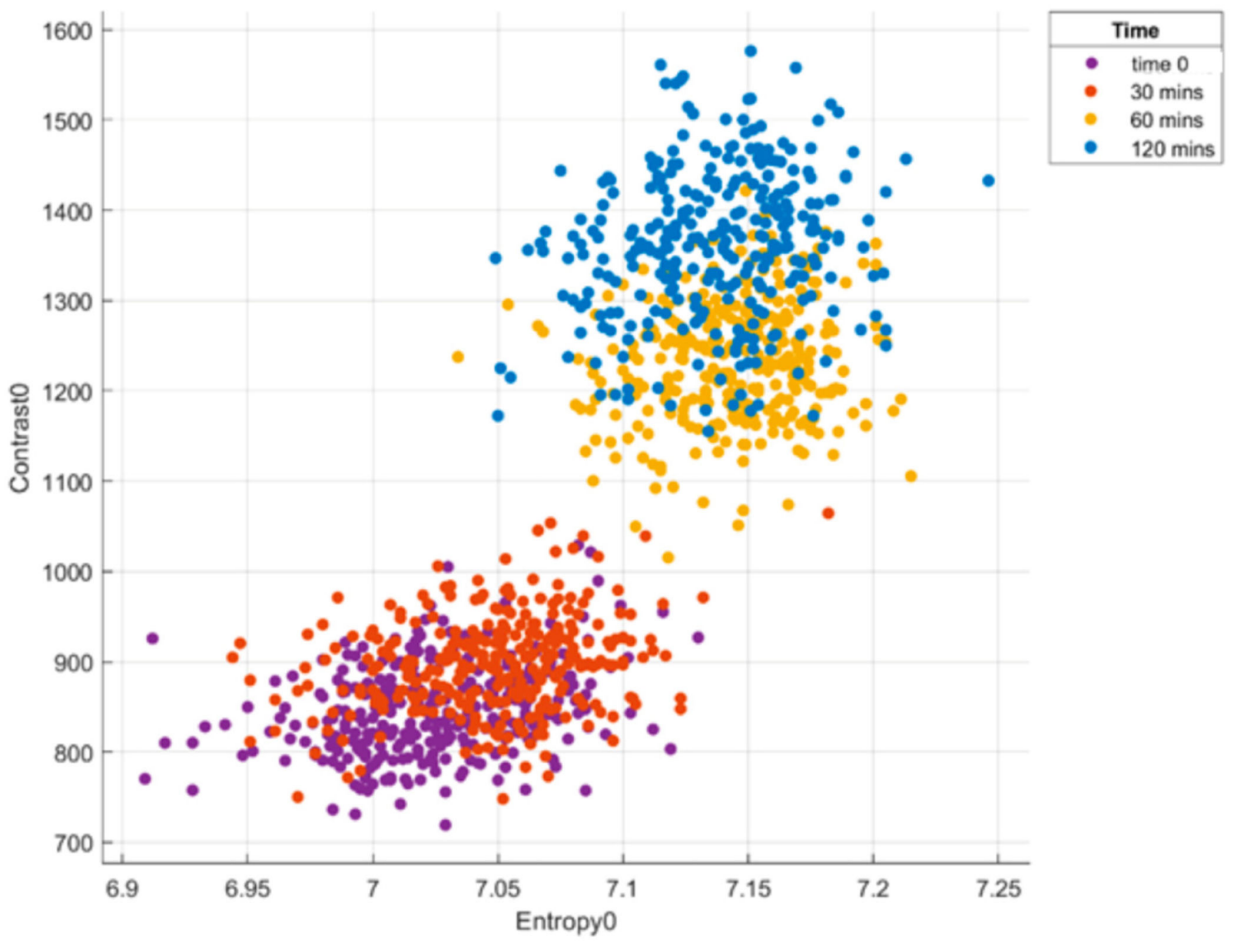
Texture analysis of post-axotomy retinal explants at the following time points: time 0, 30 min, 60 min, and 120 min. Four clusters in the feature space: the two most discriminating grey-level co-occurrence matrix features—contrast and entropy—demonstrated a better classification performance.

The layers of the murine retina were discernible throughout the experiment (Figure 4D), which allowed for the selection of VOI from the IPL and the avoidance of areas within the blood vessels. Along with the other features of the grey-level dependency matrix, during the feature selection process, the entropy and contrast in ϑ = 0° between the reference (*j*-th) and index (*i*-th) pixels provided the most benefit to the performance of the SVM classifier (Figure 5): the clusters of early (0 and 30 min) and late (60 and 120 min) stages of post axotomy were separable in the feature space. The data from all the VOI of all the retinal explants were imported into the ML classifier.

After the transection of the optic nerve, the cluster representing the images at 30 min shifted slightly right and to the top of the feature space above. In contrast, at 60 and 120 min, the clusters in the 2D feature space shifted more in the same direction from the baseline time 0 position, and the border between the early and late stages of axotomy was visible. We noted a considerable overlap at 0 and 30 min (no discrimination), but clear separation at 0 and 60 min can be seen in the 2-dimensional feature space (Figure 5) and in Table 1.

The rate of misclassification was greatest for the samples at 0 and 30 min: 24 ± 1% of the false negative rate with the SVM only and 29 ± 1% with the PCA + SVM. By contrast, the time images taken at 60 and 120 min could be classified with a high degree of accuracy (92.5 ± 0.5%). In summary, we can discriminate between time 0 and 60 min, and 30 min and 60 min, but not between time 0 and 30 min or 60 min and 120 min.

## 4 Discussion

The MTF analysis confirmed that retinal explants, in an NB medium under room air temperature maintained optical transparency 2 h after optic nerve transection during which time we could detect the changes in optical texture using OCT microscopy.

Previous work suggests that organelle fragmentation particularly among mitochondria [21] is a source of change in optical texture/contrast. However, given the numerous candidate sources of a change in retinal light backscatter [31–33], the precise identification of these scatterers lies outside the scope of this study. A recent analysis of retinal phantoms containing scattering agents with refractive indices that mimic the optical properties of organelles such as mitochondria indicates that they generate variation in optical texture that can be used to discriminate scattering agents [25].

The shift of the cluster in the later stages of apoptosis (60 and 120 min) in the 2-dimensional space (from 20D after a PCA dimensionality reduction) demonstrates a change in the optical signature aligned with the period of RGC degeneration. Similar apoptotic changes, initiated by the administration of staurosporine [34,35] were investigated in an RGC-5 cell culture for the in vitro detection of early apoptosis using an ultrahigh-resolution OCT [21,36].

The axial resolution of OCT is determined by the wavelength of the coherent light [37–40] and the full width at half maximum (FWHM) of the light source [41–44]. It is important to note that a useful time point classification could be achieved even though our device’s longitudinal and transverse resolutions are not matched (non-isotropic) [45], with the central λ = 1040 nm and FWHM = 70 nm. It is reasonable to conclude that a higher FWHM (consequently with a higher axial resolution) would further enhance the accuracy of texture-based discrimination.

## 5 Conclusions

Detecting apoptosis, particularly in its early stages, remains a topic of considerable interest and clinical relevance. The present studies further support using ligand-free methods for quantifying retinal health [13,25]. Although the present study is restricted to the retinal ganglion cell layer where axotomy can be used as a precise temporal driver of neuronal degeneration, the explant model is suited to studying the optical scattering associated with the degeneration of the outer retinal layers.

## Figures and Tables

**Table 1:** Machine learning-based classification summary.

SVM Classification							
			Predicted Class (% Correct)			TPR	FPR
	Time Points	Time 0	30 min	60 min	120 min		
**True Class**	time 0	75%	25%	0%	0%	75%	25%
	30 min	23%	77%	0%	0%	77%	23%
	60 min	0%	0%	97%	3%	97%	3%
	120 min	0%	0%	4%	96%	96%	4%
**PCA and SVM Classification**							
			**Predicted Class (% Correct)**			**TPR**	**FPR**
	**Time Points**	**Time 0**	**30 min**	**60 min**	**120 min**		
**True Class**	time 0	70%	30%	0%	0%	70%	30%
	30 min	28%	72%	0%	0%	72%	28%
	60 min	0%	0%	92%	8%	92%	8%
	120 min	0%	0%	7%	93%	93%	7%

Abbreviations: TPR—true positive rate; FPR—false positive rate.

## Data Availability

Information on the data underpinning this publication, including access details, can be found in the Figshare Research Data Repository at https://doi.org/10.6084/m9.figshare.28451531.

## Abbreviations

The following abbreviations are used in this manuscript:

### Abbreviations

ELM: External limiting membrane
ESF: Edge spread function
FFT: Fourier transform
FPR: False positive rate
FWHM: Full width at half maximum
GCL: Ganglion cell layer
GLCM: Grey-level co-occurrence matrix
HBSS: Hank’s balanced salt solution
INL: Inner nuclear layer
IPL: Inner plexiform layer
IS/OS: Junction between the photoreceptor outer and inner segments
LSF: Line spread function
ML: Machine learning
MTF: Modulation transfer function
NB: Neurobasal
OCT: Optical coherence tomography
ONL: Outer nuclear layer
OPL: Outer plexiform layer
PCA: Principal component analysis
PR: Photoreceptor
PSF: Point spread function
RGC: Retinal ganglion cell
RNFL: Retinal nerve fibre layer
ROI: Region of interest
RPE: Retinal pigment epithelium
SVM: Support vector machine
TPR: True positive rate
